# Antimalarial drug prescribing practice in private and public health facilities in South-east Nigeria: a descriptive study

**DOI:** 10.1186/1475-2875-6-55

**Published:** 2007-05-04

**Authors:** Martin Meremikwu, Uduak Okomo, Chukwuemeka Nwachukwu, Angela Oyo-Ita, John Eke-Njoku, Joseph Okebe, Esu Oyo-Ita, Paul Garner

**Affiliations:** 1 Institute of Tropical Diseases Research and Prevention, University of Calabar Teaching Hospital, Calabar, GPO Box 1211, Nigeria; 2 Department of Medical Services, Cross River State Ministry of Health Headquarters, Calabar; 3 Liverpool School of Tropical Medicine, UK

## Abstract

**Background:**

Nigeria's national standard has recently moved to artemisinin combination treatments for malaria. As clinicians in the private sector are responsible for attending a large proportion of the population ill with malaria, this study compared prescribing in the private and public sector in one State in Nigeria prior to promoting ACTs.

**Objective:**

To assess prescribing for uncomplicated malaria in government and private health facilities in Cross River State.

**Method:**

Audit of 665 patient records at six private and seven government health facilities in 2003.

**Results:**

Clinicians in the private sector were less likely to record history or physical examination than those in public facilities, but otherwise practice and prescribing were similar. Overall, 45% of patients had a diagnostic blood slides; 77% were prescribed monotherapy, either chloroquine (30.2%), sulphadoxine-pyrimethamine (22.7%) or artemisinin derivatives alone (15.8%). Some 20.8% were prescribed combination therapy; the commonest was chloroquine with sulphadoxine-pyrimethamine. A few patients (3.5%) were prescribed sulphadoxine-pyrimethamine-mefloquine in the private sector, and only 3.0% patients were prescribed artemisinin combination treatments.

**Conclusion:**

Malaria treatments were varied, but there were not large differences between the public and private sector. Very few are following current WHO guidelines. Monotherapy with artemisinin derivatives is relatively common.

## Background

The Nigerian government has recently changed its policy guidelines for treating uncomplicated malaria to artemether-lumefantrine or amodiaquine plus artesunate replacing monotherapy with chloroquine and sulfadoxine-pyrimethamine (SP). The policy change became necessary because the therapeutic efficacy of chloroquine and SP had deteriorated [1]. Any introduction of new treatments will require evidence from audit to understand current prescribing practices, and training to provide guidance [2]. The success of a new treatment policy would depend on the adherence of health providers and patients to the recommendations [3], and, in Nigeria, as in many other countries, there is a powerful pharmaceutical industry that aims to influence prescribing in both the private and public sector. Clinicians in the private sector are often thought to use more irrational treatments than in the public sector. In Nigeria, the private sector is responsible for treating over half of malaria cases [4]. To help understand the current prescribing practice, an audit of prescribing practices for malaria was conducted in both government and private facilities in Cross River State, South-eastern Nigeria.

## Methods

The study was conducted in a sample of 13 health facilities situated in rural and urban areas of Cross River State Nigeria between August and December 2003. The study area is located within the tropical rain forest belt of South-eastern Nigeria, and has an annual rainfall of 2000–3000 millimeters. Malaria transmission is intense and perennial in this area. A recent study in the area showed the therapeutic efficacy of chloroquine and sulphadoxine-pyrimethamine to be below 25% [1]. In the government sector, data were collected from the six functioning hospitals. In the private sector, permission was sought from the seven clinics and all consented. Of the total of 13 facilities, eight were urban and five rural.

In each facility, the individual records of patients recently treated for uncomplicated malaria as diagnosed by the clinician within six months of the study were audited. If no diagnosis was recorded, uncomplicated malaria was defined as fever with malaria parasitaemia in patients that were not severely unwell. A total of 30–90 records were assessed per facility depending on the out-patient attendance of each facility. Two paediatrics registrars and two medical officers were trained on the study procedure, and worked concurrently in a single team to extract data from patient record files in the selected health facilities using pre-tested data extraction forms.

Data obtained included patients age, sex, symptoms, signs, diagnostic tests and antimalarial drugs used. "Detailed history recorded" was classified when the attending clinician had in addition to a list and duration of the main symptoms, documented associated symptoms, past medical history, family and social history. There were insufficient data in the patients' records to determine the appropriateness of the treatment dosages using the drug dosage tables derived from recommendations of the national malaria treatment guideline and the World Health Organization as planned. The State Ministry of Health approved the study. The confidentiality of the patients' record and clinicians' identity were adequately protected. Data entry and analysis were with EPI-INFO 2002.

## Results

Of the 665 patient records assessed across the 13 health facilities, about half were from government services (Table 1); about half were adults. Government services were more likely to record the history or physical examination (Table 2). Malaria blood slides were only performed in 45% of patients, with no difference between private and public sector. Almost all who were screened for parasites were positive, probably because positive parasitaemia was a criterion for case definition of malaria in this study. Most cases were with a low parasitaemia.

**Table 1:** Patient characteristics and physician's assessment

**Characteristics**		**Patients (%)**		
		
		Government hospitals	Private clinics	Total
Numbers studied		348 (52.3)	317 (47.7)	665 (100)
Residence	Rural	160 (46.0)	79 (24.9)	239 (35.9)
	Urban	186 (53.4)	238 (75.1)	424 (63.8)
	Not specified	2 (0.6)	0 (0.0)	2 (0.3)
Age (years)	<5	84 (24.1)	44 (13.9)	128 (19.3)
	5+	223 (64.1)	135 (42.6)	358 (53.8)
	Not specified by clinician	41 (11.8)	138 (43.5)	179 (26.9)
Gender	Female	192 (55.2)	144 (45.4)	336 (50.5)
	Male	156 (44.8)	161 (50.8)	317 (47.7)
	Not specified	0 (0.0)	12 (3.8)	12 (1.8)
Clinical Assessment	Malaria blood smear performed	45.9% (157)	44.1% (137)	44.2% (294)
	Detailed history recorded	25.1% (86)	4.8% (15)	15.2% (101)
	General exam or one system	73.1% (250)	38.6% (120)	55.6% (370)
Malaria parasite blood slide results*	Number assessed (n)**	156	139	295
	Scanty	35 (22.4)	13 (9.3)	48 (16.3)
	+	82 (52.6)	80 (57.6)	162 (54.9)
	++	31 (19.9)	41 (29.5)	72 (24.4)
	**+++/**numerous	5 (3.2)	4 (2.9)	9 (3.0)
	Negative	3 (1.9)	1 (0.7)	4 (1.4)

* Species not specified; usually *P. falciparum *> 95% in the area.

** Percentage of number assessed (n)

**Table 2:** Prescription pattern for antimalarial drug combination and monotherapy

***Antimalarial drug regimens prescribed***	**Frequency of prescription (% n)**	
	
	Government hospitals **(n = 348)**	Private Clinics **(n = 317)**
***Monotherapy***	263(75.6)	264 (83.3)
Chloroquine	82 (23.6)	119 (37.5)
Sulphadoxine-pyrimethamine (SP)	92 (26.4)	59 (18.6)
Artemisinin	59 (16.9)	46 (14.5)
Quinine	16 (4.6)	28 (8.8)
Halofantrine	2 (0.6)	6 (1.9)
Amodiaquine	3 (0.9)	0 (0.0)

***Combination therapy***	85 (24.4)	53 (16.7)
Chloroquine-SP	35 (10.1)	16 (5.0)
Quinine-SP	32 (9.2)	24 (7.6)
Mefloquine-SP	0 (0.0)	11 (3.5)
Artemisinin-SP	2 (0.6)	2 (0.6)
Artemether+lumefantrine	16 (4.6)	0 (0.0)
Insufficient data to determine	9 (2.6)	6 (1.9)

Monotherapy was 77% of all prescriptions. The commonest drug prescribed was chloroquine (30.2%), followed by sulphadoxine-pyrimethamine (22.7%) and artemisinin alone (15.8%). There was little difference between private and government services. Combination therapy was 20.8% of all prescriptions, and commonest combination treatment was chloroquine with sulphadoxine-pyrimethamine. Only 20 patients in total received artemisinin combined with other drugs (3.0%), mostly artemether-lumefantrine in the government sector. Mefloquine-sulphadoxine-pyrimethamine was used in the private sector and formed 20.8% (11/53) of their combination treatments. There was insufficient information to determine whether 15 (2.2%) of the prescriptions were combination treatments or not.

## Discussion

This study has shown a wide variety of anti-malaria drug prescribing, but with overall prescribing in government and private facilities being remarkably similar. Monotherapy with chloroquine, sulphadoxine-pyrimethamine and artemisinin compounds were the first, second and third most common prescription practices respectively. As this audit was conducted in a period when Nigeria was transiting from chloroquine to artemisinin-based combination therapy (ACT) as recommended treatment for uncomplicated malaria, it is not surprising perhaps that this audit reflects old recommendations. What is, however, of concern was the high use of artemisinin monotherapy which was neither a recommendation in the old nor the new national treatment policy [5]. The current WHO treatment guideline for uncomplicated malaria discourages artemisinin monotherapy [6]. Irrational use of artemisinin and its derivatives as monotherapy could negate one of the goals of combination therapy which is to prevent the emergence of drug resistant malaria parasites [7,8].

A Nigerian survey of malaria control practices showed that less than a fifth of the primary and secondary health facilities studied used the recommended malaria treatment guidelines [4]. Lack of adherence to malaria treatment guidelines was associated with inappropriate prescribing practices in rural Kenya [9]. Poor drug use practices like use of sub-therapeutic doses or failing to complete prescribed doses are among factors that could lead to emergence and spread of mutant resistant strains of *Plasmodium falciparum *[7]. Poor adherence to prescriptions tends to occur more with drug regimens that have long treatment durations such as artemisinin monotherapy that takes seven days than artemisinin combination treatments that take only three days [10]. The practice of artemisinin monotherapy in this part of Nigeria if unchecked, could compromise the efficacy of artemisinin compounds.

Clarity of guidelines, strong evidence, adequate funding of guidelines and support by opinion leaders especially professional bodies are some of the factors that positively influence the use of clinical guidelines [11]. There is a need for in-depth study of factors that affect the dissemination and use of treatment guidelines in Nigeria.

The private sector is a leading provider of malaria case management in many endemic countries [12], and responsible for treating over 50% of malaria cases in Nigeria [4]. While this study showed no significant difference in prescribing practice between private and government health care service, it showed deficiencies in both sectors, and poor adherence to national and WHO guidelines. As Nigeria introduces a new treatment regimen, efforts to improve malaria treatment practices should therefore target both private and government-supported health providers. Training programmes in malaria case management should include private sector providers.

Also private-public partnership in the procurement, distribution and use of good quality anti-malaria drugs should also be encouraged to minimize the circulation of substandard drugs. Recent report of studies in some Southeast Asian countries revealed that a significant proportion of the artemisinin drugs used in those countries were substandard [13]. It is important to establish effective surveillance mechanisms to prevent the same unscrupulous suppliers from contaminating the Nigerian anti-malarial drug supply chain.

Medical record systems in the health facilities involved in this study were suboptimal with incomplete documentation of treatment details which made it difficult to assess the appropriateness of antimalarial drug dosage. Availability of modern medical record system has been identified as a key factor facilitating clinical audit [14]. Modernizing medical record systems in Nigeria will facilitate the development of clinical auditing. There is need to develop and effectively disseminate evidence-based practice guidelines as a strategy for improving prescribing practice in both public and private health care facilities.

## Competing interests

The author(s) declare that they have no competing interests.

## Authors' contributions

Uduak Okomo, Chukwuemeka Nwachukwu, John Eke-Njoku and Joseph Okebe: performed data collection, contributed to data analysis and made inputs to the manuscript; Angela Oyo-Ita and Esu Oyo-Ita: supervised data collection and contributed to writing the paper; Martin Meremikwu and Paul Garner: designed the study and drafted the paper. All the authors read and approved the final manuscript.
